# Important underground roosts for bats in Bulgaria: current state and priorities for conservation

**DOI:** 10.3897/BDJ.11.e98734

**Published:** 2023-01-25

**Authors:** Stanimira Deleva, Nia Toshkova, Maksim Kolev, Krizler Cejuela Tanalgo

**Affiliations:** 1 Universidad de Costa Rica, San José, Costa Rica Universidad de Costa Rica San José Costa Rica; 2 National Museum of Natural History at the Bulgarian Academy of Sciences, Sofia, Bulgaria National Museum of Natural History at the Bulgarian Academy of Sciences Sofia Bulgaria; 3 Institute of Biodiversity and Ecosystem Research at the Bulgarian Academy of Sciences, Sofia, Bulgaria Institute of Biodiversity and Ecosystem Research at the Bulgarian Academy of Sciences Sofia Bulgaria; 4 Ecology and Conservation Research Laboratory (Eco/Con Lab), Department of Biological Sciences, College of Science and Mathematics, University of Southern Mindanao, Kabacan, Philippines Ecology and Conservation Research Laboratory (Eco/Con Lab), Department of Biological Sciences, College of Science and Mathematics, University of Southern Mindanao Kabacan Philippines

**Keywords:** bats, Bulgaria, caves, conservation, priorities, vulnerability, BCVI, artificial roosts, mines

## Abstract

Bulgaria has a very rich bat fauna and large colonies of bats can be found in caves, mines and other underground roosts. Respectively, there are more than 107 underground roosts that are listed as important bat sites, most of which are protected by statutory laws and are of national or international importance. Despite the existence of formal protection, many roosts face anthropogenic disturbances due to the popularity of outdoor activities, such as caving and the lack of actual regulation. Currently, the evaluation was only based on the size of the colony and the presence of protected species. However, this approach is limited to roosts that contain high diversity and neglects the ones that contain high biotic importance that are highly threatened by various threats. Here, we evaluated conservation priorities and identified the most vulnerable underground bat roosts in Bulgaria, using the Bat Cave Vulnerability Index and proposed measures to adequately protect sites. We found that 32% of the Bulgarian bat roosts assessed are at a "high priority" level for conservation and protection, while 39% are at a "medium priority" that may require constant monitoring. This novel and integrative approach applied to bat roost prioritisation in the country enabled the detection of sites that need urgent conservation attention and is the first step in establishing better strategies for the bat monitoring network in Bulgaria.

## Introduction

With more than 6000 caves (Bulgarian Federation of Speleology 2022) , large areas of well-preserved natural habitats, an abundance of abandoned structures and a mild climate, Bulgaria is a suitable place for bats. Of the 47 species inhabiting Europe (IUCN 2022b), 33 are recorded in the country (Benda et al. 2003, Schunger et al. 2004, Niermann et al. 2007, Popov and Lakovski 2019, IUCN 2022a). All bat species in Bulgaria are protected by law (Republic of Bulgaria 2022). All of the 12 species listed as a priority for conservation by the Habitats Directive inhabit caves, 10 of them being considered cave-dwelling and two species are using caves during periods of swarming and hibernation (EU 1992, Ivanova 2005). Considering the enormous diversity of bats and the numerous underground roosts in the country, priority for monitoring and conserving is given to a limited number of sites that are listed as Important Bat Underground Habitats (Ivanova 2005).

The important bat underground roosts in Bulgaria were first classified by Ivanova (2005). The criteria initially used were according to the guidelines for the selection of Biological Sites of Special Scientific Interest (SSSIs) of the Nature Conservation Council in Great Britain: “4 or more species and 50 or more individuals; 3 or more species and 100 or more individuals; 2 or more species and 150 or more individuals” (Walsh et al. 2019). The previous list has included 92 underground roosts, with some of the underground roosts sheltering significant diversity of bats with regional, national or international importance (Ivanova 2005, EUROBATS 2022). The list includes caves and artificial roosts - buildings, bunkers and mines. This list was gradually updated and now consists of 107 sites, most of which are subject to regular monitoring according to the National Biodiversity Monitoring System at the Ministry of Environment and Waters of Bulgaria (Petrov 2015a, Petrov 2015b, MOEW 2022c, Toshkova and Deleva 2022). Although most of the important bat roosts are included in some form of a protected area, not all are specifically protected (e.g. the establishment of physical protection) due to the presence of important and vulnerable bat colonies in the cave site. For example, some roosts are considered natural landmarks or archaeological sites and, hence, the restrictions represent their cultural or aesthetic importance and do not necessarily consider the conservation of the biodiversity present. In addition to caves and mines, the important bat roosts in Bulgaria include several buildings and structures with environmental conditions, suitable for cave-dwelling bats, i.e. overground bat sites. Although the presence of protected bat species should guarantee the preservation of every roost (Republic of Bulgaria 2022), the conservation state of buildings, particularly those structures which are abandoned, is often uncertain. In some cases, this leaves some bat roosts more vulnerable to anthropogenic pressures than others.

Bat populations in Bulgaria are threatened by continuous habitat loss, pollution, climate change, wind turbines and disturbance and are particularly vulnerable in their roosts (Popov 2018). The existing protection of important underground roosts considers only the diversity and abundance of bats, but their susceptibility to threats and human pressures are widely neglected. In this way, there are some roosts that are mismatched with protection and persistently threatened due to their high accessibility and popularity amongst cave visitors. Other roosts, located in remote areas, are equipped with gates and signboards despite being only visited sporadically by speleologists and researchers (SFN 2020). Although often inhabited by large bat colonies, artificial roosts, such as abandoned buildings, bunkers or mines, are overlooked during conservation planning. Therefore, there is a need to establish urgent and more practical protection measures for the most vulnerable underground roosts to ensure the preservation of bat populations and their ecosystem services in the country. The Bat Cave Vulnerability Index (Tanalgo et al. 2018) is a practical tool to identify the most vulnerable caves and set priorities for future conservation. The Index integrates several important factors, such as species diversity, presence of rare species, size of colonies and morphological characteristics of caves and their approach. It was already successfully applied in several countries and artificial roosts (Deleva and Chaverri 2018, Tanalgo et al. 2022b). In this study, we applied this approach to determine the levels of conservation priorities for bat roosts in Bulgaria and to guide our focus on sites that require additional protection and urgent conservation actions. We have proposed key conservation actions for each roost, in accordance with the Conservation Evidence Initiative (Berthinussen et al. 2021). Consequently, we hope that this work would be relevant to developing effective policy-making related to the protection and conservation of important bat roosts in Bulgaria.

## Material and methods

**Figure 1.**
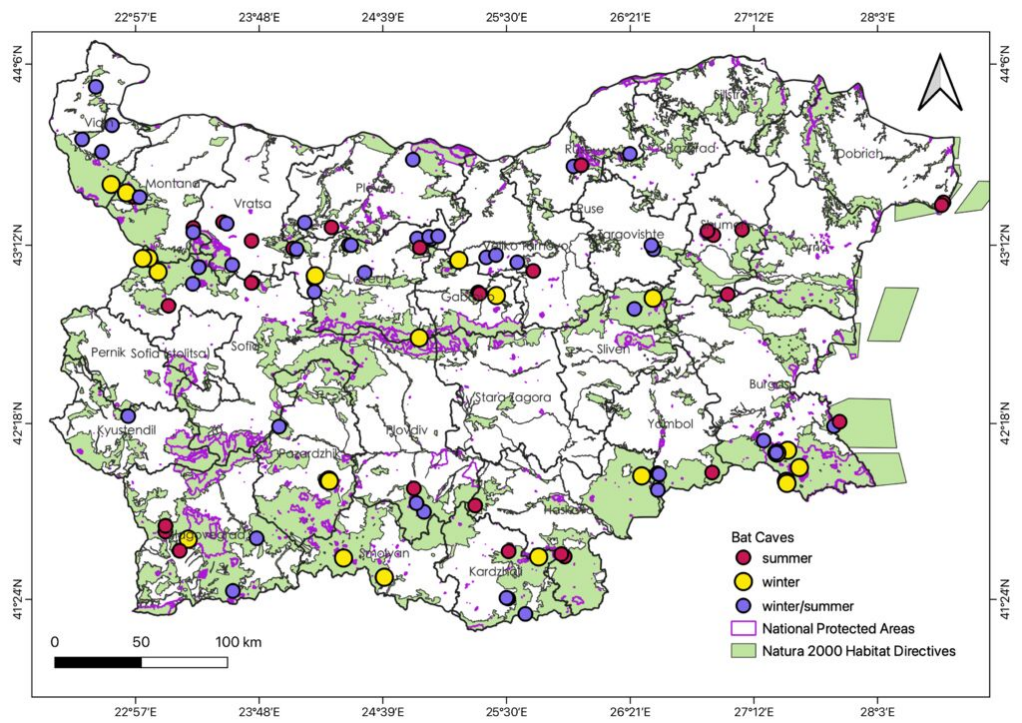
Important bat underground roosts and protected areas in Bulgaria.

The study was carried out on underground roosts and overground structures with large bat colonies located in the Republic of Bulgaria (Fig. 1). We built a dataset that includes all important underground bat roosts, following Ivanova (2005) (Suppl. material 1). Our sources are from the period between 2003 and 2022, with most of the data obtained before 2017. We obtained data for the distribution of each bat species amongst roosts and the location of each roost from the available literature, such as published research articles (Benda et al. 2003, Ivanova 2005), official monitoring reports (Petrov 2010, Petrov 2015a, Toshkova and Deleva 2022), the database of the Natura 2000 network in Bulgaria available at the website of the Ministry of Environment and Waters, i.e. MOEW (2022a) and the national database of the National Biodiversity Monitoring System (available upon request at MOEW (2022c)). We checked the conservation state of each roost using the information on protected areas of Bulgaria (MOEW 2022b). We checked if a roost is located within one or more protected areas using the spatial data provided by the Ministry of Environment and Waters (MOEW 2022d). When a roost was located in overlapping protected areas, for example - a Natural landmark and a Natura 2000 zone, we took into account the higher level of protection or the one with restrictions on visits.

### Assessing conservation priority using BCVI

We assessed cave priorities using the Bat Cave Vulnerability Index (BCVI) (see Tanalgo et al. (2018) for a complete prioritisation scheme). The index is composed of two components: Biotic Potential (BP) and Biotic Vulnerability (BV). The Biotic Potential (BP) takes into account the bat roost species richness, abundance, relative abundance, endemism and conservation status. We report the abundance of each species as the maximum number of individuals observed at each roost. The Biotic Vulnerability (BV) assesses the characteristics of the cave landscape feature and threats, such as cave morphology, visitation and land use in the surrounding areas. As the Index was originally developed for tropical caves, we adapted new criteria to assess the Biotic Vulnerability (BV) score that is contextualised in the Bulgarian environment. For example, in Bulgaria, cave temples are rare, but some of our caves shelter industrial structures, such as dairy farms, places to grow mushrooms, fuel repositories and wine cellars (Bulgarian Federation of Speleology 2022). We consequently changed the category from “temples” to “temples and structures” and included the following categories: 4 = no structures are present, 3 = old and abandoned structures are present, 2 = structures may be present, but rarely used (e.g. water-capturing structures, that are maintained several times a year), 1 = functioning and frequently-used structures (e.g. operating dairies, mushroom gardens, temples, wine cellars etc.) are present. The BP Index has a value between 1 and 4, with 1 being the highest level of priority. The BV Index has values of A, B, C and D, with A being the most vulnerable to disturbances. The sub-indices (BP and BV) are integrated to obtain the BCVI status and determine the overall priority of the caves. We used the latest IUCN Red List (version 2022-1) for the assessment of each species' global conservation and endemism status. In addition to the BCVI, we present new data on the importance status of each roost, following the methodology used in Bulgaria up to now, described by Ivanova (2005). The categories of importance are based on the presence of the total number of individuals and the number of species in each roost: Regional (25 to 100 individuals of ≥ 4 species), National (100 to 500 individuals of ≥ 3 species or 500 to 1000 individuals of ≥ 2 species) and International (1000 or more individuals of ≥ 2 species). We did all calculations in Excel 2021 for Windows (Microsoft corporation 2021). We mapped caves and their conservation status using the software QGIS v. 3.26 (QGIS 2022) and visualised data using R Studio (R Studio Team 2021).

### Assessing suitable conservation actions

In addition to the Vulnerability Index, we assessed the condition and existing potential threats to each roost, based on the physical signs present, for example, collapsed entrances, household waste, graffiti and broken infrastructure (Petrov 2015b). We used the latest monitoring reports and the database of the National Museum of Natural History as a source of information (Petrov 2010, Petrov 2015a, Toshkova and Deleva 2022). We conducted an intensive literature search to effectively develop and propose appropriate conservation actions for each specific site. We used the available data from the Conservation Evidence initiative (Berthinussen et al. 2021) and considered the general assessment of each conservation action, the individual study used in its evaluation in combination with all the guidelines and recommendations provided by the Eurobats working groups. Then we measured their relevance for our specific cases and species. We selected only effective bat conservation actions with high-quality evidence and no undesirable effects.

## Results

All the 33 bat species and six species complex groups found in Bulgaria were evaluated for all underground sites. According to the IUCN Red List (IUCN 2022a), the majority of the species are considered as Least Concern (n = 27),and six are Near Threatened (n = 6). There are three (3) species under the threatened category (Vulnerable) and a single data-deficient species. The Bulgarian Red Data Book (Golemanski et al. 2015) lists as Least Concern 11 species, as Near Threatened four species, 10 species are listed as Vulnerable, two species are data-deficient and six species do not have an assigned category. The most common species include *Rhinolophusferrumequinum*, which occurs in at least 89% (n = 96 sites) of cave sites, followed by *Rhinolophushipposideros* (73%, n = 75 sites) and *Miniopterusschreibersii* (70%, n = 75 sites). Several species were not observed in underground roosts or were very rare as they are not considered cave-dwelling (n = 7). We present the species of bats, their conservation status and distrubuition in roosts in Table 1.

**Figure 2.**
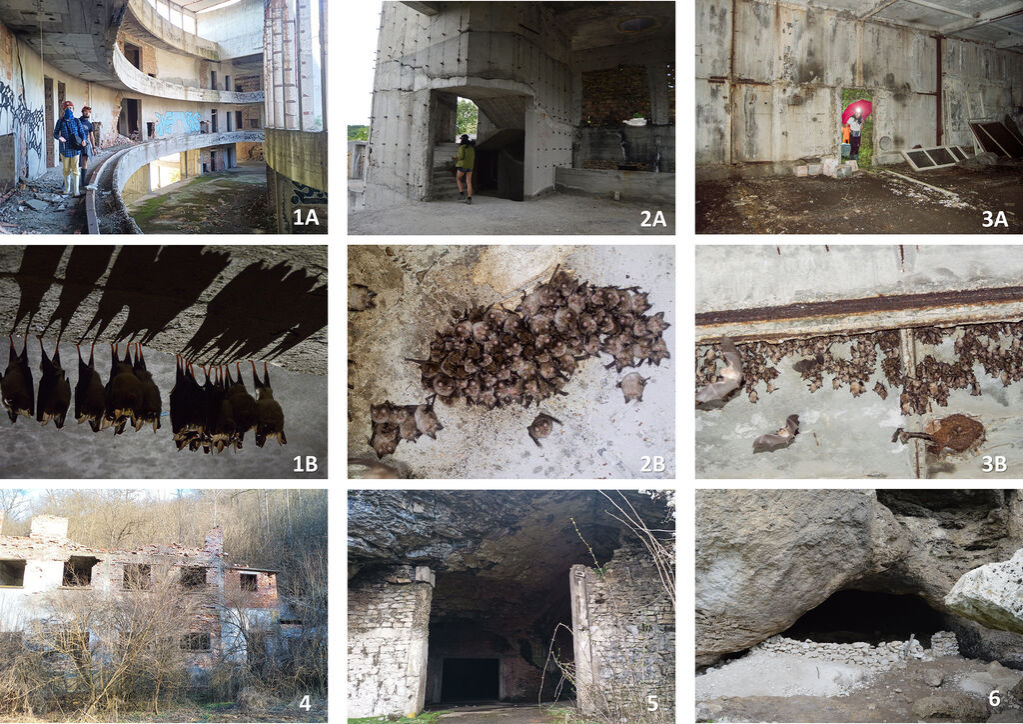
Artificial structures such as Perla 2 (1A and 1B), Abandoned residency (2A and 2B) and an abandoned mushroom greenhouse (3A and 3B) are sheltering large colonies of cave-dwelling bats. Some natural caves in Bulgaria are adapted for human use: Mandrata in the village of Mikre (4) has a whole house built at the entrance, the cave with the same name nearby - Mandrata at Alexandrovo, is accessible with an automobile (5). The Karangin Cave, located in the Rhodope Mountain is turned into a sheepfold (6). Photo credit: S. Deleva.

**Figure 3.**
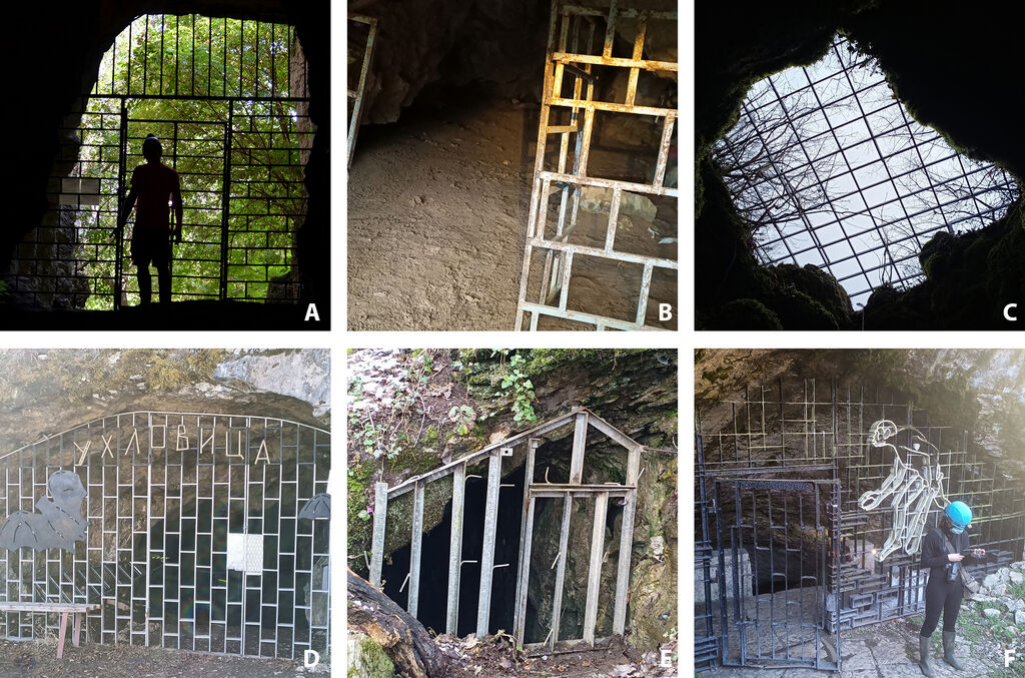
Improper gate design: A - the gate at the Musinska Cave allows bats to fly in and out, but it is not optimal. B - the cave at the entrance of Kalna Matnitsa Cave remains open to allow bat access. C - The Kaleto Cave entrance is equipped with a gate, that might stop bats, but does not stop visitors. D - The gate at the Uhlovitsa show Cave is still waiting for its renovation. E - Although the intention of the gate at the Bratanova Cave entrance is to protect bats, it is built without consulting with the EUROBATS recommendations. F - The gate at one of the entrances of the Magurata show cave. Photo credit: S. Deleva.

**Figure 4.**
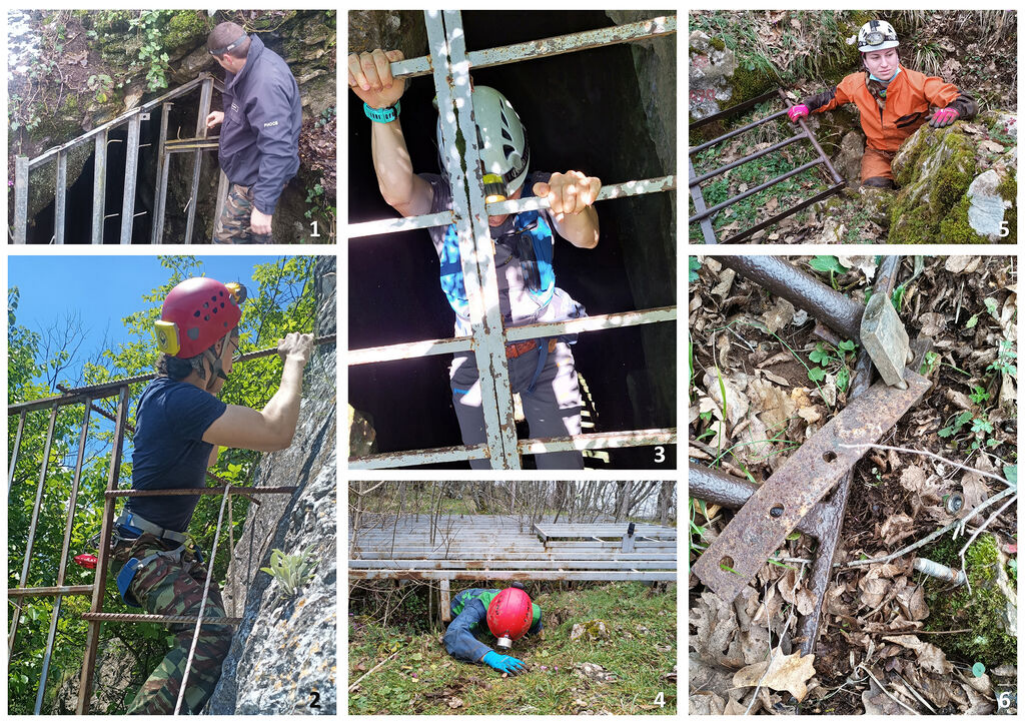
Examples of ineffective cave gates: 1 - Parts of the gate of the Bratanovata Cave are twisted to allow easier access. 2. The fence at the Divdyadovski Zandan Cave cannot stop visitors. 3 - Access to the Derventskata Cave is officially restricted, but cavers are freely passing through the gate. 4 - Kaleto Cave has a locked gate, but cavers have created a shortcut under it. 5 and 6 - the Elenina dupka Cave has a very strong gate, but cavers have unscrewed the bolts holding the padlock. Photo credits: S. Deleva (1, 2, 4 and 6), M. Kolev (3), S. Markova (5).

We assessed a total of 107 underground sites for this current prioritisation analysis. We obtained data for 92 bat roosts from previous records (Ivanova 2005). We included an additional 15 sites recently added to the list, with 96 (90%) natural caves, six (6%) overground sites (buildings, Fig. 2), three (3%) mine sites and two (2%) bunkers. We included information on location, occupancy (summer, winter or both), protected areas, importance, threats and species diversity. The exact coordinates of the roosts could not be shared publicly as the locations contain the presence of sensitive to disturbance species and habitats, for which visitation, even for research purposes, could be harmful. We present the low-resolution coordinates of the roosts in Suppl. material 1, following the recommendations of the Best Practices for Generalising Sensitive Species Occurrence Data (Chapman 2020). The exact locations will be made available upon request. Regarding the level of protection, most of the sites (n = 64) received legal protection in the form of visitation prohibition by the Natura 2000 network (Habitats Directive), 31 cave sites are located in protected natural landmarks, nine caves within protected areas and two within natural reserves (Suppl. material 1). A single cave (Tangarachkata) does not have legal protection. Almost all the roosts were subjected to some form of visitation regulations. Visitation is prohibited during the breeding season of bats (from 1 March to 30 June) in 54 sites, a single site during the hibernation period (from 1 December to 31 March) and both breeding and hibernation periods in three sites. Visitation is prohibited all year round in 28 sites and three caves are restricted for camping or group visits. There are no visitation restrictions for 13 sites. Physical conservation actions and restrictions present in Bulgaria include gates, fences, signs and some security regulations. There are 18 sites currently equipped with gates and seven have a fence around the entrance. Signboards with information about bats are placed on 37 sites. There are six show caves with more strict protection due to their economic value (e.g. entrance gate, opening hours, personnel and signalling security equipment) (Suppl. material 1). The disturbance is by far the main concern for the majority of the sites (n = 98), followed by the threat of roost destruction (n = 4) and improper gate design (n = 4, Fig. 3). Only one site did not face any conservation concerns, as the bat colony is located in a heavily guarded area. The main target groups, which might cause disturbance are tourists (n = 35), cavers (n = 55, Fig. 4), rock climbers (n = 1) and occasional visitors (n = 1). In eight of the sites, the main disturbing factors were cave and bat researchers, who were the most frequent visitors. Our suggested conservation actions include restriction of visitation, modification of cave gates, placement of signboards and actions, specifically aimed at cavers. In the case of the Tangarachkata cave, we propose that the site should be declared a protected area.

**Figure 5.**
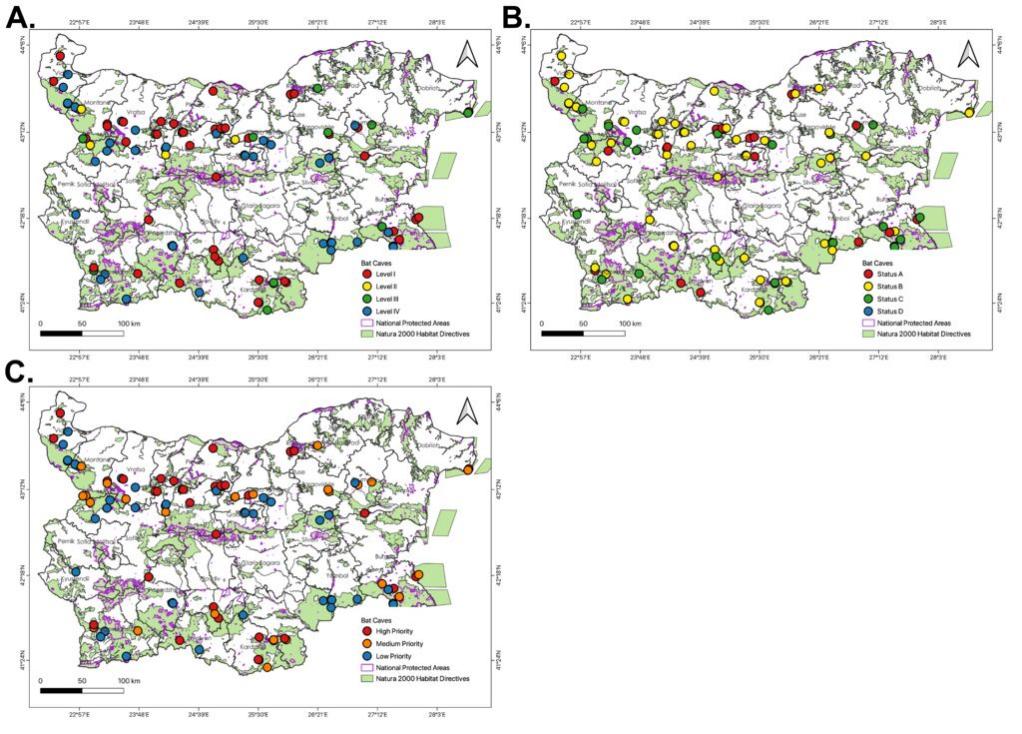
Underground roosts priorities according to (A) Biotic Potential (BP), (B) Biotic Vulnerability (BV) and (C) BCVI priorities.

**Figure 6.**
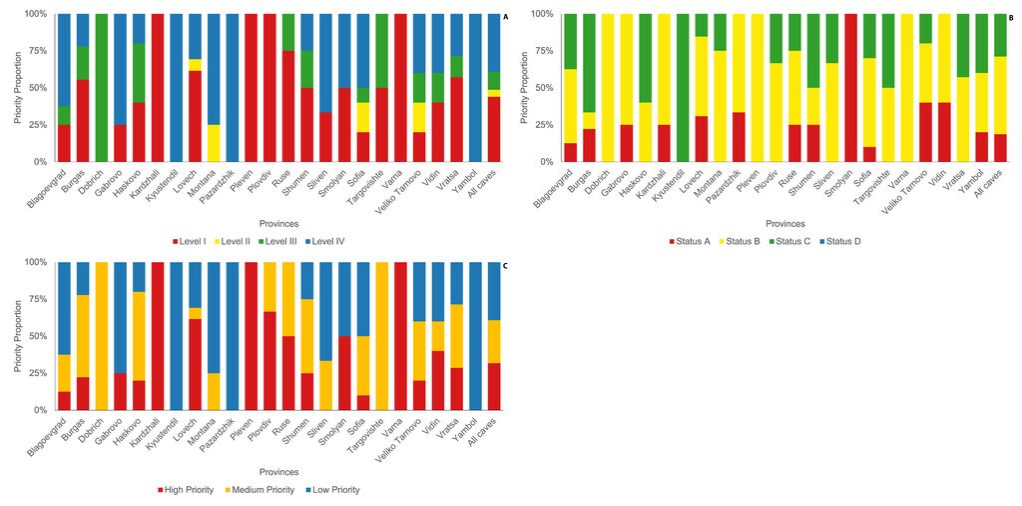
Comparison of (A) Biotic Potential (BP), (B) Biotic Vulnerability (BV) and (C) BCVI priorities, across provinces.

We used data generated from a previous cave assessment over a period of time for our BCVI prioritisation (Table 2). Therefore, our results for species diversity and abundance represent the maximum population estimates of each roost rather than the current state of the populations. Amongst the assessed caves in terms of Biotic Potential (BP), 47 (44%) of the caves have the highest BP (Level 1), while five (5, 5%) caves at mid-high (Level 2), 13 (12%) caves at mid-low (Level 3) and 42 (39%) roost at the lowest level (Level 4). In terms of Biotic Vulnerability (BV), 20 (19%) of the sites were the most vulnerable to threats (Status A), 56 (52%) are in the mid-high vulnerability (Status B) and 31 (29%) are in the mid-low level. No cave sites were recorded in Status D (i.e. the lowest level of vulnerability). Of the roosts with the highest BP, 43 are natural caves and four are buildings and infrastructures. Five of the most vulnerable bat roosts (Status A) are show caves, but three are not, yet they are as easy to explore and even more accessible than a show cave. There were 31 roosts that scored as low conservation priority. Most of them are vertical caves, located in remote areas with restricted access (Table 2). At the provincial level, BP levels did not show a significant difference (χ² = 77.41, p = 0.1591) with four (n = 4) and two (n = 2) provinces having all its roosts considered in high and low levels in terms of BP, respectively. Similarly, BV did not show a significant difference at the provincial level (χ² = 45.10, p = 0.426). Only a single province has all its caves falling within high vulnerability. Overall, combining BP and BV, we identified 34 (32%) high-priority caves that require the highest and most urgent need of conservation protection and 42 (39%) bat caves mid-priority that may need monitoring to ensure the existing population continues to thrive, while there are 31 (29%) at low priority, which can be potentially considered for other cave use and activities due to the absence of important or vulnerable bat populations (Fig. 5). When compared at the provincial level (χ² = 249.515, p = 0.083), three Provinces (Kardzhali, Pleven and Varna) have all caves assessed as high-priority for conservation, while single provinces have all caves in medium-priority (Dobrich) and low-priority (Yambol) (Fig. 6). All threats and conservation actions are presented in Suppl. material 1. When we used the criteria, described by Ivanova (2005), the importance status of the roosts was the following: International - 61 roosts, National - 33 roosts, regional - 6 roosts, no status - 7 roosts (Suppl. material 1).

## Discussion

Caves and underground habitats are important for at least 48% of global bat species, with 28% of bat caves being threatened, but information on key priority caves for conservation at the national level remains limited (Tanalgo et al. 2022a). This work is the first effort to comprehensively assess the vulnerability and conservation priorities of important bat roosts in Bulgaria and their protection. Since its implementation as part of the integration of the country into the European Union, the Natura 2000 network covers more than 30.3% of the territory of Bulgaria and caves are listed as habitats of community interest (code 8310) (EU 1992). Most important bat roosts are included either as separate protected zones, i.e. declared for the protection of a single cave or as a part of a larger protected area. All bat species in Bulgaria are legally protected, with 12 species, most of which are cave-dwelling, listed in Annex II of the Habitats Directive. Any form of visitation, including touristic activities and speleological exploration are restricted in most protected areas where habitat 8310 - "Caves not open to the public" or bat species are listed as objects of conservation priority for the Habitats Directive. The Natura 2000 network is proven effective at covering territories with natural caves and the presence of cave-dwelling bats (Lisón et al. 2013), but implementing its regulations is not optimal in Bulgaria as we often observe threats, such as unregulated visitation in protected sites . In reality, most of the important bat roost sites face disturbance and other anthropogenic threats, such as polution and vandalism (Toshkova and Deleva 2022).

Cave visitation restriction is by far the most effective conservation action, but its application in Bulgaria has proven to be very difficult. The proposed period of visitation restriction for most of the sites (1 March to 30 June) does not match the period of the actual breeding season for bats and their occupancy in the roost and this is concerning, especially in the conditions of the changing climate that might affect the roosting patterns amongst bats (Festa et al. 2022). Visitation restrictions, especially the regimens of the Natura 2000 network, are not enforced in practice and often cavers and tourists are unaware of or ignore the existing regulations. The most vulnerable roosts (Status A) need urgent conservation actions with individually chosen conservation interventions. The most extreme restriction measures, such as cave gating, may have mixed and even negative effects on bat populations (Mitchell-Jones et al. 2007, Berthinussen et al. 2021) and should be applied with caution and after carefully considering all existing evidence. If physical restrictions to the entrance are needed, we recommend a fence around a large perimeter and not a gate, in accordance with the recommendations of the EUROBATS agreement (Mitchell-Jones et al. 2007). Moreover, the blocking of cave entrances with objects or vegetation should be avoided and actions for clearing entrances should be a priority in future conservation projects.

One of the issues in conservation is that the nature protection legislation prioritises the natural caves (EU 1992). However, due to different factors, such as habitat loss, many bats are increasingly roosting in artificial structures, whose conservation status remains unclear or absent. Some of the most important roosts for bats in Bulgaria are artificial structures, for example, buildings, mines and even one operating structure in a dam. These structures have already been established as important habitats for bats and some of them have been occupied for many years (Ivanova 2005). In some countries, the establishment of artificial underground roosts for bats is practised as part of conservation initiatives. Still, in Bulgaria, artificial structures are often neglected by decision-makers. To our knowledge, there are no buildings declared as protected sites due to the presence of bats in Bulgaria. Moreover, those roosts are particularly vulnerable as they could naturally collapse or face destruction by the owners. For example, the abandoned hotel "Perla 2" is currently sheltering thousands of bats from 11 different species. However, this building is a private property and there are projects for demolition (Melteva 2013). The MOEW should consider these abandoned buildings as overground bat roosts and adopt the accepted conservation measures of underground sites for their protection.

Show caves are important bat roosts, but are excluded from the 8310 habitat and from monitoring obligations. Attention and efforts towards show caves as important bat roosts and their inclusion as a habitat of importance when considering monitoring and conservation initiatives should be necessary (Weigand et al. 2022). In our assessed caves, often only part of the show cave is accessible to tourists. For example, less than a kilometre of the area of Orlova Chuka Cave is open for visitors, but the only entrance is locked due to its show cave status. This leads to a limited access to the rest of the cave - more than 13 km of galleries are protected from disturbance. A positive example of a show cave in Bulgaria that considers bats is the Biserna (Zandana) Cave in the Shumensko Plato Nature Park. The Cave is open for controlled visitation only during spring and autumn and the entrance is locked during the hibernation and breeding seasons. Cave tourism is often a double-edged sword in a way that it could affect bat cave biodiversity by disrupting bat behaviour and their roosting habitat (Furey and Racey 2016). Still, properly-managed cave tourism could potentially promote bat conservation and cave protection (Debata 2020, Tanalgo and Hughes 2021).

Caves that were considered less vulnerable using the index (BV, Status B and C), are caves that require effort to access, such as special equipment, high exploration efforts, permits or are located in remote areas. Cavers, researchers and, in rare cases, tomb raiders, are often the key factors contributing to the disturbance in these roost sites. Our assessment shows that the efforts to physically protect caves, located in remote areas are likely ineffective in protecting bat colonies. We observed that reinforcement of regulations is often ignored by many visitors, evident by the removal of signboards and damage to existing gates (Toshkova and Deleva 2022, Fig. 4). Speleology is popular in Bulgaria and imposing rules that are impossible to enforce would only lead to conflicts. However, when properly trained, cavers could potentially be part of effective conservation measures by engaging them in bat conservation and monitoring (Bücs 2020). Anthropogenic disturbance to cave bats is not the only pressure that threatens cave biodiversity, but may potentially be exacerbated by other threatening processes, such as habitat loss, pesticides and climate change. Concentrating efforts on increasing awareness amongst cavers and local people should be prioritised and integrated with conservation initiatives in cave protection in the country. As the climate in Bulgaria has been changing in recent years (Marinova et al. 2017, Dale and Zhekova 2019), bat colonies are expected to move to more suitable roosts; thus, the need to focus efforts towards identifying and monitoring vulnerable sites are equally significant initiatives. Speleologists in Bulgaria could contribute significantly to filling the knowledge gaps in bat distribution if given the opportunity. A solution to minimise disturbance would be to provide an evidence-based visitation protocol.

The Bat Cave Vulnerability Index (BCVI) was originally developed for the prioritisation of bat caves in the tropical region (e.g. in Deleva and Chaverri (2018)). Using appropriate metrics and components to assess cave priorities, the Vulnerability Index enables identifying areas with high conservation importance. This is the first extensive application of the approach outside the tropical realm and has shown effectiveness in identifying underground sites for conservation, including artificial ones. This Index provides an alternative to the approach to identify the roost importance, based solely on diversity and abundance (Ivanova 2005) and the two methods could be compared and their reliability evaluated once we have more data. The prioritisation has certain limitations, for example, the influence of seasonality on bat abundances and species cave occupation, sampling methods and efforts, which definitely would alter the biotic potential of caves and the overall priorities of caves. Although these caveats require future validation and testing, our current work provides a useful overview of bat cave conservation in Bulgarian subterranean habitats. In our analysis, we found that 18% of the important bat roosts are currently facing a severe level of threat that requires immediate action. These caves are a high priority for both the research effort and monitoring, while mid-priority caves need to be monitored to ensure that remaining populations are protected from further declines. Consequently, the priorities set for caves will be relevant to inform policy-makers to effectively protect bats and other organisms dependent on healthy underground ecosystems.

## Conclusions

Our current work has demonstrated the prioritisation of important underground roosts for conservation and protection and has discussed key issues and threats in them. Here, we found that anthropogenic activities, such as widespread caving activities and tourism, are the main concerns for bat roosts in the country, particularly in sites such as Mandrata (Alexandrovo), Emenskata, Perla 2, Rezidentsia Shumen, Karangin and Suhi Pec. We urge decision-makers to prioritise the sites that require urgent conservation attention to preserve important bat populations. We have also found that, while the Natura 2000 network is effective in covering the important bat roosts, the regulations are not well enforced on many sites. Most of the important bat sites in Bulgaria are legally protected by the Natura 2000 network and their visitation is prohibited all year or during specific periods. Yet, most of the sites are imperilled by severe disturbance combined with other threats. The existing restrictions, especially in the case of the national protected area network, need to be updated to specifically address bats and to reflect the current state of the roosts. Furthermore, using a novel integrative approach for prioritisation, we were able to identify vulnerable and important underground roosts for conservation in Bulgaria. We have also shown the feasibility and effective use of such an approach in the European context, which may be a useful step forward to the application of the Index in European caves through the adaptation of conservation organisations (e.g. Eurobats). We hope that our current work would inspire more effort by developing policies to protect cave-dwelling bats and their roosts in the country, especially in the face of the changing human environment.

## Supplementary Material

811B7C99-CF40-5811-87D7-5B4FACF3A62D 10.3897/BDJ.11.e98734.suppl1 Supplementary material 1 Important underground bat roosts in Bulgaria Data type dataset Brief description Important underground bat roosts in Bulgaria, their location, conservation status, importance, Bat Cave Vulnerability Index, bat biodiversity and visitation restrictions. File: oo_797263.xlsx https://binary.pensoft.net/file/797263 Stanimira Deleva, Nia Toshkova, Maxim Kolev, Krizler Tanalgo

## Figures and Tables

**Table 1.** Cave-dwelling bats recorded in Bulgaria, their roost distribution and conservation status. The cave-dwelling species are marked with *. No of caves: the number of roosts from the current dataset in which the species is observed. Relative occurrence: the relative occurrence of the bat species in all caves assessed in the study. IUCN: Conservation status according to IUCN Red List (Global). BG Red List: Conservation status, according to the Bulgarian Red Data Book (Golemanski et al. 2015). BBA: Appendices of the Bulgarian Biodiversity Act. 92/43 EEC: Appendices of the COUNCIL DIRECTIVE 92/43/EEC of 21 May 1992 on the conservation of natural habitats and of wild fauna and flora. BERN: Berne Convention on the Conservation of European Wildlife and Natural Habitats. BON: Appendices of the Convention on the Conservation of Migratory Species of Wild Animals. EUROBATS: the species is listed in the EUROBATS agreement for the conservation of the populations of the European bats.

**Code**	**Species**	**No of caves**	**Relative occurrence**	**IUCN**	**BG Red Book**	**BBA**	**92/43 ЕЕС**	**BERN**	**BON**	**EUROBATS**
Rhifer	Rhinolophusferrumequinum*	96	89.72	LC	NT	2, 3	2, 4	II	II	+
Rhihip	Rhinolophushipposideros*	78	72.897	LC	LC	2, 3	2, 4	II	II	+
Minsch	Miniopterusschreibersii*	75	70.093	NT	VU	2, 3	2, 4	II	II	+
Rhieur	*Rhinolophuseuryale*	74	69.159	NT	VU	2, 3	2, 4	II	II	+
Myomyo/bly	*Myotismyotis*/ M.blythii*	66	61.682	LC	NT	3	4	II	II	+
Myobra	*Myotisbrandtii*	55	51.402	LC	LC	3	4	II	II	+
Myocap	Myotiscapaccinii*	54	50.467	VU	VU	2, 3	2, 4	II	II	+
Myobly	Myotisblythii*	45	42.056	LC	NT	2, 3	2, 4	II	II	+
Myomyo	Myotismyotis*	44	41.121	LC	NT	2, 3	2, 4	II	II	+
Myoema	Myotisemarginatus*	41	38.318	LC	VU	2, 3	2, 4	II	II	+
Rhimeh	Rhinolophusmehelyi*	26	24.299	VU	VU	2, 3	2, 4	II	II	+
Rhimed	*Rhinolophusmedia* species complex*	25	23.364	LC/NT	VU	2, 3	2, 4	II	II	+
Pleaus	*Plecotusaustriacus*	25	23.364	LC	LC	3	4	II	II	+
Rhi sp.	*Rhinolophus* sp.*	24	22.43	N/A		2, 3	2, 4	II	II	+
Myobec	*Myotisbechsteinii*	24	22.43	NT	VU	2, 3	2, 4	II	II	+
Rhibla	Rhinolophusblasii*	23	21.495	LC	VU	2, 3	2, 4	II	II	+
Myodau	*Myotisdaubentonii*	23	21.495	LC		3	4	II	II	+
Eptser	*Eptesicusserotinus*	21	19.626	LC	LC	3	4	II	II	+
Nycnoc	*Nyctalusnoctula*	16	14.953	LC	LC	3	4	II	II	+
Hipsav	*Hypsugosavii*	16	14.953	LC	LC	3	4	II	II	+
Pippip	*Pipistrelluspipistrellus*	14	13.084	LC	LC	3	4	II	II	+
Myonat	*Myotisnattereri*	13	12.15	LC	LC	3	4	II	II	+
Barbar	*Barbastellabarbastellus*	12	11.21	NT	VU	2, 3	2, 4	II	II	+
Myo sp.	*Myotis* sp.	9	8.4112	N/A	N/A	3	4	II	II	+
Pleaur	*Plecotusauritus*	8	7.4766	LC	NT	3	4	II	II	+
Myomys	*Myotismystacinus*	8	7.4766	LC	LC	3	4	II	II	+
Vesmur	*Vespertiliomurinus*	6	5.6075	LC	LC	3	4	II	II	+
Myoalc	*Myotisalcathoe*	5	4.6729	DD		3		II	II	+
Nyclei	*Nyctalusleisleri*	5	4.6729	LC	VU	3	4	II	II	+
Pippyg	*Pipistrelluspygmaeus*	5	4.6729	LC		3	4	II	II	+
Rhimeh/eur	*Rhinolophusmehelyi*/ R.euryale*	4	3.7383	N/A	VU	2, 3	2, 4	II	II	+
Myoaur	*Myotisaurascens*	4	3.7383	LC		3	4	II	II	+
Pip sp.	*Pipistrellus* sp.	3	2.8037	N/A		3	4	II	II	+
Pipkuh/nat	*Pippistrilluskuhlii*/ *P.nathusii*	3	2.8037	LC		3	4	II	II	+
Pipkuh	*Pipistrelluskuhlii*	3	2.8037	LC		3	4	II	II	+
Pipnat	*Pipistrellusnathusii*	3	2.8037	LC	LC	3	4	II	II	+
Tadten	*Tadaridateniotis*	3	2.8037	LC	DD	3	4	II	II	+
Eptnil	*Eptesicusnilssonii*	0	0	LC	DD	3	4	II	II	+
Myodas	*Myotisdasycneme*	0	0	NT		3	2, 4	II	II	+
Nyclas	*Nyctaluslasiopterus*	0	0	VU	VU	3	4	II	II	+
Ple sp.	*Plecotus* sp.	0	0	N/A	N/A	3	4	II	II	+

**Table 2.** Important bat underground roosts in Bulgaria and the Bat Cave Vulnerability Index. The show caves are marked with *.

BP	BV	Type	Name	Occupancy	Legal visitation restrictions	Main concern	Target group	Immediate conservation actions
1	A	Cave	Devetashkata Peshtera*	Winter/Summer	Show cave	Disturbance	Tourists	Daily security. Signboards. Fines.
1	A	Cave	Dyavolskoto Garlo*	Winter	Show cave		Tourists	Not needed
1	A	Cave	Emenskata Peshtera	Winter/Summer	Breeding	Disturbance	Tourists	Physical restriction of access to the cave entrance.
1	A	Cave	Karangin	Summer	Year-round	Disturbance	Tourists	Signboards
1	A	Cave	Magurata*	Winter/Summer	Show cave	Disturbance	Tourists	Light reduction
1	A	Cave	Mandrata (Chavdarci)	Winter/summer	Breeding	Disturbance	Tourists	Physical restriction of access to the cave entrance.
1	A	Cave	Orlova Chuka*	Winter/Summer	Show cave	Disturbance	Researchers	Limitation of bat capturing
1	A	Building	Perla 2	Winter/Summer	No	Destruction	Owners	Immediate protection
3	A	Bunker	Bunker Gara Peyo Yavorov	Summer	Breeding	Disturbance	Tourists	
3	A	Cave	Musina (Musinskata)	Winter/Summer	No	Disturbance	Tourists	
3	A	Cave	Suhi Pech	Winter/Summer	No	Disturbance	Cavers	Targeted at cavers
4	A	Cave	Bacho Kiro	Winter	Year-round	Improper gate design	Tourists	Modification of the gate
4	A	Cave	Futiovata Peshtera	Winter/Summer	Breeding	Disturbance	Tourists	Signboards
4	A	Cave	Leyarnitsite	Winter/Summer	Camping	Disturbance	Tourists	
4	A	Cave	Razkopkite	Summer	Breeding	Disturbance	Tourists	Physical restriction of access to the cave entrance.
4	A	Building	Rezidentsia Shumen	Summer	Breeding	Destruction	Owners	Immediate protection
4	A	Cave	Saeva Dupka	Winter	Year-round	Disturbance	Tourists	
4	A	Cave	Snezhanka*	Winter	Show cave	Disturbance	Tourists	
4	A	Cave	Uhlovica*	Winter	Year-round	Improper gate design	Local authorities	Modification of the gate
4	A	Cave	Vodnata	Winter/Summer	Breeding	Disturbance	Cavers	Targeted at cavers
1	B	Cave	Aina Ini	Winter/Summer	Year-round	Disturbance	Researchers	Restriction of visitations by the local RIEW
1	B	Cave	Andaka	Winter/Summer	Breeding	Disturbance	Cavers	Targeted at cavers
1	B	Cave	Bilernicite	Summer	Breeding	Disturbance	Tourists	Daily security. Signboards. Fines.
1	B	Cave	Biserna (Zandana)*	Winter/Summer	Breeding	Disturbance	Tourists	Not needed
1	B	Cave	Elenina Dupka	Winter	Year-round	Disturbance	Cavers	Targeted at cavers
1	B	Building	Gabarnitsi Bachkovo	Summer	Breeding	Collapse	Occasional visitors	Physical restriction of access to the cave entrance - Fence
1	B	Cave	Gargina Dupka	Winter/Summer	Breeding and hibernation	Disturbance	Cavers	Targeted at cavers
1	B	Mine	Golashkata Peshtera	Winter/Summer	Year-round	Disturbance	Researchers	Immediate protection
1	B	Cave	Haydushkata Peshtera (Devenci)	Winter/Summer	Breeding	Disturbance	Cavers	Targeted at cavers
1	B	Building	Kresnenski Prolom - Zhp Kanton	Summer	Breeding	Disturbance	Tourists	
1	B	Cave	Mandrata (Mikre)	Winter/Summer	Breeding	Disturbance	Tourists	Physical restriction of access to the cave entrance.
1	B	Cave	Nanin Kamak	Winter/Summer	Breeding and hibernation	Disturbance	Tourists	Immediate protection
1	B	Cave	Parnicite - Dolniya Parnik	Winter/Summer	Year-round	Disturbance	Cavers	Targeted at cavers
1	B	Cave	Parnicite - Gorniya Parnik	Winter/Summer	Year-round	Disturbance	Cavers	Targeted at cavers
1	B	Cave	Ponora	Winter/Summer	Year-round	Disturbance	Cavers	Targeted at cavers
1	B	Building	Rezervoari Madzharovo	Summer	Breeding	Destruction	Owners	Immediate protection
1	B	Cave	Samara	Winter/Summer	Year-round	Disturbance	Tourists	
1	B	Cave	Sedlarkata	Summer	Year-round	Disturbance	Cavers	Targeted at cavers
1	B	Cave	Skoka	Summer	Breeding	Disturbance	Cavers	Targeted at cavers
1	B	Cave	Tauk Liman	Summer	Breeding	Disturbance	Tourists	
1	B	Cave	Troevratica (Zidanka)	Winter/Summer	Breeding	Disturbance	Cavers	Targeted at cavers
1	B	Cave	Varkan	Winter/Summer	Year-round	Disturbance	Cavers	Targeted at cavers
1	B	Cave	Vodnite Dupki	Winter	Year-round	Disturbance	Tourists	
1	B	Cave	Yarasa Ini	Summer	Breeding	Disturbance	Researchers	Limitation of visits
1	B	Cave	Urushka Maara	Winter/Summer	Breeding	Disturbance	Tourists	
1	B	Cave	Zorovica	Summer	Breeding	Disturbance	Researchers	Limitation of visits
2	B	Cave	Dinevata Pesht	Winter	Year-round	Disturbance	Cavers	Targeted at cavers
2	B	Cave	Morovica	Winter/Summer	Year-round	Disturbance	Cavers	Targeted at cavers
2	B	Cave	Razhishkata	Winter/Summer	Breeding	Disturbance	Tourists	
2	B	Building	Tunnels in the wall of Al. Stamboliyski Reservoir	Winter	No	None	None	Not needed
3	B	Cave	Bozhkova Dupka	Winter/Summer	Year-round	Disturbance	Cavers	Targeted at cavers
3	B	Cave	Chelovechata	Summer	Breeding	Disturbance	Cavers	Targeted at cavers
3	B	Cave	Marina Dupka	Winter/Summer	Year-round	Disturbance	Cavers	Targeted at cavers
3	B	Cave	Tyulenovata Peshtera (Sv.N)	Summer	Breeding	Disturbance	Cavers	Targeted at cavers
4	B	Cave	Bashovichki Pec	Winter/Summer	Group activities only	Disturbance	Cavers	Targeted at cavers
4	B	Cave	Bozhiyat Most	Summer	Year-round	Disturbance	Tourists	Better signboards
4	B	Cave	Bozkite	Winter	Breeding	Disturbance	Tourists	Remove the gate
4	B	Cave	Golyamata Mitrovska	Summer	Breeding	Disturbance	Tourists	Physical restriction of access to the cave entrance.
4	B	Cave	Golyamata Prilepna	Summer		Disturbance	Tourists	
4	B	Cave	Kolibata	Summer	Breeding	Disturbance	Cavers	Targeted at cavers
4	B	Cave	Kozarnika	Summer	Breeding	Disturbance	Tourists	Physical restriction of access to the cave entrance.
4	B	Bunker	Kresnenski Prolom Bunker	Summer	Breeding	Disturbance	Tourists	
4	B	Mine	Lesovo Galerii	Winter/Summer	No	Disturbance	Tourists	
4	B	Mine	Minna Galeria Aida	Summer	Breeding	Disturbance	Tourists	
4	B	Cave	Mishin Kamak	Winter	Year-round	Disturbance	Cavers	Targeted at cavers
4	B	Cave	Novata (Starata) Peshtera	Winter	Breeding	Disturbance	Cavers	Targeted at cavers
4	B	Cave	Orlovata Peshtera	Winter/Summer	Breeding	Disturbance	Tourists	
4	B	Cave	Padaloto (Izvorat Na Yantra)	Summer	No	Disturbance	Cavers	Targeted at cavers
4	B	Cave	Prileparnika	Winter/Summer	No	Disturbance	Cavers	Targeted at cavers
4	B	Cave	Sharaliyska Peshtera	Winter	Hibernation	Disturbance	Cavers	Targeted at cavers
4	B	Cave	Starshelitsa	Winter/Summer	Breeding	Disturbance	Cavers	Targeted at cavers
4	B	Cave	Subatta	Winter	Year-round	Disturbance	Cavers	Targeted at cavers
4	B	Cave	Temnata Dupka (S. Milanovo)	Winter/Summer	Year-round	Disturbance	Cavers	Targeted at cavers
4	B	Cave	Tsarskata	Winter/Summer	Breeding	Disturbance	Cavers	Targeted at cavers
4	B	Cave	Vodni Pech	Winter	Breeding	Disturbance	Cavers	Targeted at cavers
4	B	Cave	Yubileyna*	Winter	Show cave	Improper gate design	Local authorities	Modification of the gate
1	C	Cave	Bratanovata Peshtera	Winter	Year-round	Improper gate design	Local authorities	Modification of the gate
1	C	Cave	Derventskata Peshtera	Winter/Summer	Year-round	Disturbance	Cavers	Targeted at cavers
1	C	Cave	Divdyadovski Zandan	Summer	Breeding	Disturbance	Climbers	Signboard inside the cave
1	C	Cave	Gabarnika	Winter/Summer	Breeding	Disturbance	Researchers	Immediate protection
1	C	Cave	Golyamata Balabanova	Winter	Breeding	Disturbance	Cavers	Targeted at cavers
1	C	Cave	Golyamata Vapa	Winter	No	Disturbance	Cavers	Targeted at cavers
1	C	Cave	Gyurgen Dere	Summer	Breeding	Disturbance	Cavers	Not needed
1	C	Cave	Ivanova Voda	Winter/Summer	Breeding	Disturbance	Cavers	Targeted at cavers
1	C	Cave	Kalna Matnica	Winter/Summer	Breeding	Disturbance	Cavers	Targeted at cavers
1	C	Cave	Lednika (Kotel)	Winter/Summer	Year-round	Disturbance	Cavers	Targeted at cavers
1	C	Cave	Manuilovata	Winter/Summer	Breeding	Disturbance	Cavers	Targeted at cavers
1	C	Cave	Serapionovata Peshtera	Winter/Summer	Breeding and hibernation	Disturbance	Cavers	Targeted at cavers
1	C	Cave	Tyulenovata Peshtera (M. Nos)	Summer	No	Disturbance	Cavers	Targeted at cavers
2	C	Cave	Parasinskata Propast	Winter/Summer	Breeding	Disturbance	Cavers	Targeted at cavers
3	C	Cave	Hilyadite Ochichki	Summer	Year-round	Disturbance	Researchers	Restriction of access to the bat colony.
3	C	Cave	Kaleto	Winter	Camping	Disturbance	Cavers	Targeted at cavers
3	C	Cave	Rupata	Winter/Summer	Breeding	Disturbance	Cavers	Targeted at cavers
3	C	Cave	Shamaka	Winter	Breeding	Disturbance	Cavers	Targeted at cavers
3	C	Cave	Tangarachkata Dupka	Winter/Summer	No	Disturbance	Cavers	Targeted at cavers
3	C	Cave	Zandana	Winter	Breeding	Disturbance	Researchers	Limitation of visits
4	C	Cave	Asandeliya	Winter/Summer	No	Disturbance	Cavers	Targeted at cavers
4	C	Cave	Dranchi Dupka	Winter/Summer	Breeding	Disturbance	Cavers	Signboard
4	C	Cave	Genchovata Peshtera	Summer	Breeding	Disturbance	Cavers	Targeted at cavers
4	C	Cave	Golyamata Vitanovska	Winter	No	Disturbance	Cavers	Targeted at cavers
4	C	Cave	Kalenska Peshtera	Summer	No	Disturbance	Tourists	
4	C	Cave	Kanchova Varpina	Summer	Breeding	Disturbance	Cavers	Targeted at cavers
4	C	Cave	Kesedzhiisa	Winter/Summer	Breeding	Disturbance	Cavers	Targeted at cavers
4	C	Cave	Lyastovicata (Glozhene)	Winter/Summer	Breeding and hibernation	Disturbance	Cavers	Targeted at cavers
4	C	Cave	Vodnata Pesht	Summer	Breeding	Disturbance	Cavers	Targeted at cavers
4	C	Cave	Zandana (Ilindentsi)	Winter/Summer	Breeding	Disturbance	Tourists	Limitation of visits
4	C	Cave	Zandana (Ruykova)	Summer	Breeding	Disturbance	Cavers	Targeted at cavers
